# A nested-PCR with an Internal Amplification Control for the detection and differentiation of *Bartonella henselae *and *B. clarridgeiae*: An examination of cats in Trinidad

**DOI:** 10.1186/1471-2334-5-63

**Published:** 2005-08-12

**Authors:** Joanne N Rampersad, John D Watkins, Michael S Samlal, Raymond Deonanan, Shalini Ramsubeik, David R Ammons

**Affiliations:** 1 Dept. of Life Sciences, The University of the West Indies, St. Augustine, Trinidad, Trinidad and Tobago; 2 School of Veterinary Medicine, The University of the West Indies, Eric Williams Medical Sciences Complex, Mt. Hope, Trinidad, Trinidad and Tobago

## Abstract

**Background:**

*Bartonella *species are bacterial blood parasites of animals capable of causing disease in both animals and man. Cat-Scratch Disease (CSD) in humans is caused mainly by *Bartonella henselae *and is acquired from the cat, which serves as a reservoir for the bacteria. A second species, *B. clarridgeiae *is also implicated in the disease. Diagnosis of Bartonellosis by culture requires a week or more of incubation on enriched media containing blood, and recovery is often complicated by faster growing contaminating bacteria and fungi. PCR has been explored as an alternative to culture for both the detection and species identification of *Bartonella*, however sensitivity problems have been reported and false negative reactions due to blood inhibitors have not generally been addressed in test design.

**Methods:**

A novel, nested-PCR was designed for the detection of *Bartonella henselae *and *B. clarridgeiae *based on the strategy of targeting species-specific size differences in the 16S-23S rDNA intergenic regions. An Internal Amplification Control was used for detecting PCR inhibition. The nested-PCR was utilized in a study on 103 blood samples from pet and stray cats in Trinidad.

**Results:**

None of the samples were positive by primary PCR, but the Nested-PCR detected *Bartonella *in 32/103 (31%) cats where 16 were infected with only *B. henselae*, 13 with only *B. clarridgeiae *and 3 with both species. Of 22 stray cats housed at an animal shelter, 13 (59%) were positive for either or both species, supporting the reported increased incidence of *Bartonella *among feral cats.

**Conclusion:**

The usefulness of a single PCR for the detection of *Bartonella henselae *and *B. clarridgeiae *in the blood of cats is questionable. A nested-PCR offers increased sensitivity over a primary PCR and should be evaluated with currently used methods for the routine detection and speciation of *Bartonella henselae *and *B. clarridgeiae*. In Trinidad, *B. henselae *and *B. clarridgeiae *are the predominant species in cats and infection appears highest with stray cats, however *B. clarridgeiae *may be present at levels similar to that of *B. henselae *in the pet population.

## Background

*Bartonella *are fastidious, gram-negative, bacteria comprised of at least 19 species and 3 subspecies [1] that are obligate parasites of the blood in reservoir animals. *Bartonella *species are considered emerging zoonotic pathogens [2] and may be involved in a number of disease presentations including angiomatosis [3] and ocular manifestations [4]. Similarly, *Bartonella *species are being associated with disease in their animal hosts (see reviews [2,5]. The role of cats as a reservoir for human Bartonellosis is well documented however probably incomplete. Studies suggest that other *Bartonella *species, known to cause disease in humans, are found in the cat (see for example [6] and [7]. Of these, *Bartonella henselae *and, to a lesser extent, *Bartonella clarridgeiae *are known to cause Cat-Scratch Disease (CSD) in humans [8]; see also [9] for review of CSD.

As a fastidious organism, *Bartonella *usually requires over a week of incubation for primary isolation. The slow growth of the organism complicates its isolation since faster growing bacteria and fungi can overrun the plate. Thus various types of tests using Polymerase Chain Reaction (PCR) have been explored as a diagnostic tool for the detection and identification of *Bartonella *species from blood [10-12]. Previously, Jensen et al., [13] developed a PCR for the detection of *Bartonella *that targets species-specific size differences in the 16S-23S rDNA intergenic region. However, as a primary PCR, it was questionable if the sensitivity of the test was optimal for the detection of relatively low numbers of bacteria [14] and a control for the detection of false-negative reactions due to inhibition by blood components was not addressed. Herein we describe the development of a nested-PCR for the detection of *B. henselae *and *B. clarridgeiae *based on the strategy of species-specific size differences in the 16S-23S rDNA intergenic region that includes an Internal Amplification Control for PCR inhibitors. The test was evaluated on the blood of 103 apparently healthy cats in Trinidad to investigate the presence of these organisms in the local cat population and to verify the test's ability to detect these organisms in the blood of apparently healthy animals.

## Methods

### Specimen collection

All samples were collected over an 11 month period in 2001. Blood samples were collected in commercial blood collection tubes containing EDTA and transported to the laboratory on ice, where possible the same day, or stored at 4°C until transported. Samples were collected from geographically distinct areas in Trinidad including an animal shelter, private veterinary clinics and the Veterinary Hospital located at the University of the West Indies' School of Veterinary Medicine by both Veterinarians and final year veterinary students.

### PCR

DNA was extracted from whole blood according to the method of Boom et al., [15] with modifications as described by Rampersad et al., [16]. One microlitre of blood-extracted DNA template was used in the primary PCR reaction and 1 μl of the primary reaction was used in the nested reaction. In order to minimize contamination, separate rooms were used for preparing the PCR reaction mix, template preparation, gel electrophoresis, and performing nested reactions. Nested reactions were performed in a biosafety hood where 1 μl of primary reaction was delivered to a nested tube with a 1 μl urine loop, the end of the loop was then washed briefly in a 10% bleach solution, rinsed in water then flamed before reusing.

Primary and nested-PCR reactions were optimized by testing 1.5, 2 and 3 mM MgCl_2 _in the reaction, each at 12 temperatures ranging from 45°C to 65°C using an Eppendorf Gradient Master Cycler. *B. henselae *and *B. clarridgeiae *strains used for template were a gift from Dr. Jill Clarridge. Optimized PCR cycle conditions were 94°C -15 s, 48.2°C -30 s and 72°C -30 s for 40 cycles for the primary-PCR and 94°C -15 s, 56°C -30 s and 72°C -30 s for 40 cycles for the nested-PCR. The PCR strategy was an adaptation of a primary PCR described by Jensen et al., [13]. Primary PCR primers were P-bhenfa (5'-TCTTCGTTTCTCTTTCTTCA) and P-benr1 (5'-CAAGCGCGCGCTCTAACC) which gave an approximately 186 bp fragment for *B. henselae *and a 168 bp fragment for *B. clarridgeiae*, and nested primers N-bhenf1a (5'-GATGATCCCAAGCCTTCTGGC) and N-bhenr (5'-AACCAACTGAGCTACAAGCC) which gave an approximately 152 bp fragment for *B. henselae *and a 134 bp fragment for *B. clarridgeiae*. The Internal Amplification Control (IAC) consisted of a DNA amplicon, randomly amplified from the genome of a chicken with primer IAC (5'-TGTTTGACAGCTTATCAT). All reactions were performed in a 25 μl volume as follows: Primary reaction (0.2 mM each dNTP, 0.5 pmoles/μl each P-bhenfa and P-benr1, 3 mM MgCl_2 _reaction buffer, 0.4 pmoles/μl primer IAC, 1 μl of IAC template stock (concentration unknown), 0.5 units *Taq *polymerase (Promega) and Nested (0.2 mM each dNTP, 0.5 pmoles/μl each N-bhenf1a and N-bhenr, 1.5 mM MgCl_2 _reaction buffer, 0.4 pmoles/μl primer IAC, 0.5 units *Taq *polymerase).

The *B. clarridgeiae*-specific PCR was performed using 1 μl of a primary reaction in a PCR reaction with primers bclarF (5'- GCACAAGCCTCTGAGAGGGA) and N-bhenr. Reactions were performed in a 25 μl volume as follows: Primary reaction (0.2 mM each dNTP, 0.5 pmoles/μl each primer, 3 mM MgCl_2 _reaction buffer, 0.5 units *Taq *polymerase). PCR cycle conditions were 94°C -15s, 55°C -30 s and 72°C -30 s for 40 cycles.

PCR amplification products were separated on either a 2% or 3% agarose gel, stained with ethidium bromide and visualized under 302 nm ultra-violet light. DNA markers were created by PCR amplification of virulence genes from *Escherichia coli *generating calculated-sized fragments (bp) of 881, 520, 337, 275 171 and relative sizes confirmed using a commercial DNA size standard (100 bp ladder, Promega).

### Culture

One hundred microliters of blood was spread on a Brain Heart Infusion Agar plate (Difco) supplemented with 10% whole sheep blood and incubated for 2–3 weeks at 37°C in 5% CO_2_. In some cases the blood was frozen at -70°C before being cultured.

### DNA sequencing

Amplification products generated in the nested reaction for *B. henselae *and *B. clarridgeiae *were electrophoresed through a 1% agarose gel. The agarose containing the amplified DNA was cut from the gel, briefly frozen then thawed and spun at 12,000 rpm through a polypropylene fibre to remove the DNA-containing liquid. The DNA was concentrated by ethanol precipitation and used in the sequencing reaction. An ABI Big Dye sequencing kit was used according to manufacturer's instructions using the nested primers individually in two separate reactions, and the reactions resolved on an ABI 377 DNA sequencing machine.

## Results

**Figure 1:**
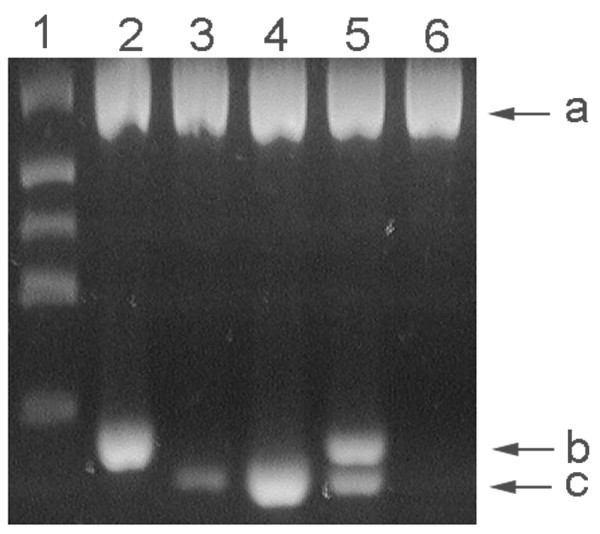
**PCR amplification products**. A 3% agarose gel showing lane 1) DNA size markers (bp) of 881, 520, 337, 275 171 and lanes 2–6) nested-PCR reactions of blood samples. Band a) internal amplification control, band b) *B. henselae*, and band c) *B. clarridgeiae*.

Of 103 blood samples subjected to PCR, none of the samples were positive for *Bartonella *in the primary reaction, but 32 were positive in the nested reaction. Inhibition was observed in 3 primary reactions indicated by a relatively light band seen for the IAC. However, one of these reactions was later positive for both *B. henselae *and *B. clarridgeiae *with the nested reaction. Two amplification bands of differing mobilities were observed in the nested reaction, both migrating below the 171 bp size marker (Figure 1). The larger fragment co-migrated with an amplification product generated from *B. henselae *(data not shown) and the smaller band was of a size expected from *B. clarridgeiae*. When the primary PCR reaction was used as template in a PCR reaction with a primer specific for *B. clarridgeiae*, only those samples that had the smaller sized fragment were positive. The identity of the respective amplification fragments were further investigated by DNA sequencing wherein the larger fragment was confirmed to be from *B. henselae *(GenBank: [DQ000494]) and the smaller-sized fragment from *B. clarridgeiae *(GenBank: [DQ003029]).

The number of *Bartonella*-negative samples along with those positive for either or both *Bartonella *species is given for both pet and stray animals in Table 1. Where the age of cats was known, infected cats (not including those from the shelter) ranged from 2–72 months. For stray cats at the animal shelter of unknown age, one half (11) of the cats were classified as adults and the other 11 as kittens, of which 6 of the 13 infected animals were adults and 7 kittens.

**Table 1:** *Bartonella *species associated with living environments of cats

	Pets	Animal shelter	Total
*B. henselae*	7	9	16
*B. clarridgeiae*	10	3	13
Both species	2	1	3
Bartonella negative	62	9	71
Total	81	22	103

The number of *Bartonella*-negative samples along with those positive for either or both *Bartonella *species is given for both pet and stray animals.

Blood cultures were generally contaminated with both spreading bacteria and fungi, to such a degree that it was felt that little meaningful data could be gained through culture, and it was discontinued.

## Discussion

As an emerging pathogen known for causing disease in both humans and animals, a simple test for the routine identification of *Bartonella *species is desirable. Because multiple *Bartonella *species are associated with disease in both humans and animals, it would be advantageous to have a test capable of both detecting different species and differentiating between them. The strategy first presented by Jensen et al., [13] generally fits these requirements by targeting DNA-length heterogeneity in the 16S rDNA-23S rDNA intergenic region. As such, this test is well suited for the detection of *B. henselae *and *B. clarridgeiae*, the causative agents of CSD in humans. However, as a single PCR reaction, this test has questionable sensitivity and has in fact been reported to show less sensitivity than culture for the detection of *B. henselae *and *B. clarridgeiae *in blood, [14]; see also [17]. Similarly, the primary reaction used in this study was unable to detect *Bartonella *DNA in any of the samples although we were able to show the presence of *Bartonella *DNA in 32 samples with a nested reaction. These results question the usefulness of a single PCR reaction for the detection of *Bartonella *in blood without further enhancing the overall sensitivity of the test, such as with the addition of a nested-PCR reaction. However, many laboratories are reluctant to use a nested reaction for fear that its high sensitivity will detect contaminating DNAs (mainly amplicons) and deliver a false-positive result. In this work, none of our negative controls were positive. To help control cross contamination, template preparation, assembly of the PCR reaction, nested reactions and gel electrophoresis were performed in different rooms. Especially useful was the use of a UV hood and a 1 μl urine loop instead of barrier tips to transfer an aliquot of the primary reaction to the nested reaction. By immersing the end of the loop, after each transfer, in a 10% bleach solution to chemically degrade any amplicons, and then rinsing with water before flaming, any chance splattering or aerosolization of DNAs from the loop during flaming was reduced.

The test of Jensen et al., [13] also lacked an Internal Amplification Control (IAC) to detect PCR inhibition. In fact, arguments have been made for Internal Amplification Controls to be mandatory when describing diagnostic tests based on PCR [18]. Since blood, which is a known source of PCR inhibitors, is a commonly used sample for *Bartonella *detection, it is important that PCR-based tests for *Bartonella *include an IAC. Individuals interested in evaluating this method may write the authors for an aliquot of the IAC template.

Because of the slow growth and long culture time for *Bartonella*, faster growing contaminating bacteria and fungi can overrun a culture plate and make the detection of *Bartonella *difficult or even impossible. Early in this study we found that the contamination problem was so severe that culture was discontinued. The unexpectedly high level of contamination may have originated from the blood collection process where blood was collected by both private veterinary clinics and veterinary students as part of their studies, and thus was not strictly controlled. Although perhaps undesirable for the purposes of this study, the uncontrolled nature of its collection perhaps more accurately represented blood as it would arrive to the laboratory for diagnosis, and underscores one advantage of PCR over culture for the detection of *Bartonella*.

Prevalence studies reported for *Bartonella *infection in cats are generally characterized by a relatively high prevalence of *B. henselae *compared to *B. clarridgeiae *[19-22]. In this study, our results were mostly consistent with other studies where *B. henselae *was detected in slightly more samples than *B. clarridgeiae*. However approximately 40% of the infected cats originated from an animal shelter, which were not necessarily representative of the cat population as a whole in Trinidad. For example, discounting the animals from the shelter, 7 (8.6%) animals were positive for *B. henselae*, 10 (12.3%) for *B. clarridgeia*e and 2 (2.5%) for both bacteria, suggesting that *B. clarridgeia*e may be found among pet cats at levels similar to, or even higher than that for *B. henselae*. As has been reported in other studies [21,22], cases of mixed infections with both species were found. In addition, there was a clear relationship between cats expected to be exposed to fleas (e.g. stray cats at an animal shelter), and the prevalence of infection.

**Figure 2:**
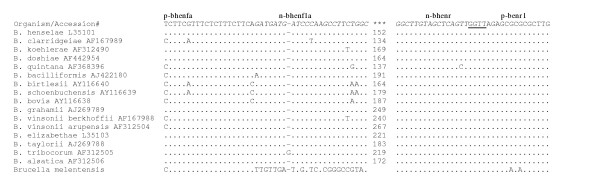
**Primer and template sequence homology**. Bacterial species are followed by their GenBank accession numbers. Primer sequences are given below the primer's name at the top, primary primers are in bold, nested primers are in italics and sequence shared by two primers is underlined. A dot (.) below each nucleotide in the primer represents homology at that position in each species' DNA with the primer and letter signifies the diverging nucleotide found in the bacteria DNA. A dash (-) represents spaces placed in the sequence for alignment purposes. Three asterisks (***) signifies the variable length region of DNA found between the forward and reverse primers and the number given at that site for each species indicates the size of the amplicons generated from the nested reaction.

Although this test was designed for the detection of *B. henselae *and *B. clarridgeiae*, sequence homology between the primers and other *Bartonella *species indicates that this test might be useful for the detection of other *Bartonella *species (Figure 2). Of the 15 species for which sequence data was available, only three species, *B. bovis, B. schoenbuchensis and B. birtlesii *would not be expected to amplify due to a single base pair mismatch between the template and the terminal 3' position of the primer. Admittedly, our test would benefit from a redesign of the forward primers (Figure 2), so that they would recognize all known *Bartonella *species, [23]. Jensen et al., [13] had identified sequence homology in the 16S-23S rRNA intergenic region between *Bartonella *with *Brucella *species. In this test, sequence homology indicates that the primary PCR should amplify *Brucella *DNA, however no such amplification should occur in the nested reaction (Figure 2).

## Conclusion

The usefulness of a single PCR for the detection of *B. henselae *and *B. clarridgeiae *in the blood of cats is questionable and enhancement methods, such as a nested-PCR, should be utilized. In Trinidad, *B. henselae *and *B. clarridgeiae *are found in cats and infection appears highest among stray cats, however *B. clarridgeiae *may be present at levels similar to, or higher than, that of *B. henselae *in pet cats.

## Competing interests

The author(s) declare that they have no competing interests.

## Authors' contributions

JNR and DRA provided project and diagnostic design/development and performed diagnostics, JDW conceived the project and provided project design and management, MSS performed template preparation and sample management, RD and SR collected blood and information on the animals.

## Pre-publication history

The pre-publication history for this paper can be accessed here:


